# Antimicrobial Stewardship in Public-Sector Hospitals in KwaZulu-Natal, South Africa

**DOI:** 10.3390/antibiotics11070881

**Published:** 2022-06-30

**Authors:** Sarentha Chetty, Millidhashni Reddy, Yogandree Ramsamy, Vusi C. Dlamini, Rahendhree Reddy-Naidoo, Sabiha Y. Essack

**Affiliations:** 1 Department of Pharmacy and Pharmacology, Faculty of Health Sciences, University of the Witwatersrand, Johannesburg 2193, South Africa; 2 Discipline of Pharmaceutical Sciences, School of Health Sciences, Westville Campus, University of KwaZulu-Natal, Durban 4041, South Africa; 3 Essential Medicine Consulting, Durban 4400, South Africa; millireddy@gmail.com; 4 Department of Medical Microbiology, Prince Mshiyeni Memorial Hospital-National Health Laboratory Services, Antimicrobial Research Unit, University of Kwazulu-Natal, Durban 4041, South Africa; ramsamyy@ukzn.ac.za; 5 Pharmaceutical Services, Department of Health, Kwazulu-Natal, Pietermaritzburg 3201, South Africa; vusi.dlamini@kznhealth.gov.za (V.C.D.); rahendhree.reddy@kznhealth.gov.za (R.R.-N.); 6 Antimicrobial Research Unit, College of Health Sciences, University of KwaZulu-Natal, Durban 4041, South Africa; essacks@ukzn.ac.za

**Keywords:** antimicrobial stewardship, South Africa, situational analysis, KwaZulu-Natal

## Abstract

Antimicrobial resistance (AMR) is a serious global public-health threat. Evidence suggests that antimicrobial stewardship (AMS) is a valuable tool to facilitate rational antibiotic use within healthcare facilities. A cross-sectional situational analysis using a questionnaire was conducted to determine the current status of antimicrobial stewardship (AMS) activities in all public-sector hospitals in KwaZulu-Natal (KZN). The survey had a 79% (57, N = 72) response rate. A total of 75% of hospitals had an antimicrobial stewardship committee (AMSC), 47% (20, N = 43) had a formal written statement of support from leadership, and 7% (3, N = 43) had budgeted financial support. Only 37% (16, N = 43) had on-site or off-site support from a clinical microbiologist, and 5% (2, N = 43) had an on-site infectious disease (ID) physician. Microbiologist input on pathogen surveillance data (aOR: 5.12; 95% CI: 4.08–22.02; *p*-value = 0.001) and microbiological investigations prior to the commencement of antibiotics (aOR: 5.12; 95% CI: 1.08–42.01; *p*-value = 0.041) were significantly associated with having either on- or off-site microbiology support. Respondents that had a representative from microbiology on the AMSC were significantly associated with having and interrogating facility-specific antibiograms (*P* = 0.051 and *P* = 0.036, respectively). Those facilities that had access to a microbiologist were significantly associated with producing an antibiogram (aOR: 4.80; 95% CI: 1.25–18.42; *p*-value = 0.022). Facilities with an ID physician were significantly associated with having a current antibiogram distributed to prescribers within the facility (*P* = 0.010) and significantly associated with sending prescribers personalized communication regarding improving prescribing (*P* = 0.044). Common challenges reported by the facilities included suboptimal hospital management support; a lack of clinicians, pharmacists, nurses, microbiologists, and dedicated time; the lack of a multidisciplinary approach; low clinician buy-in; inadequate training; a lack of printed antibiotic guidelines; and financial restrictions for microbiological investigations. The survey identified the need for financial, IT, and management support. Microbiology and infectious disease physicians were recognized as scarce human resources.

## 1. Introduction 

Antimicrobial resistance (AMR) is a serious global public-health threat [1,2,3,4,5]. A recent report revealed that drug resistance accounted for 1.2 million deaths, globally [6]. The development of antibiotic resistance in bacteria is a natural phenomenon; however, indiscriminate use of antibiotics has exacerbated and perpetuated AMR [1,3,4]. The loss of effective antibiotics threatens the outcome of modern-day surgery, cancer chemotherapy, organ transplant, and the treatment of infectious diseases [1,7]. In addition to patient care, resistance also impacts the local and global economy. The treatment of infections caused by multi-drug resistant (MDR) bacteria requires longer and more expensive therapeutic regimens coupled with additional diagnostic testing, translating to increased costs to patients and the healthcare system [1,8].

AMR is a multifaceted problem requiring urgent intervention using multiple strategies [1,2,7]. Surveillance, infection prevention and control (IPC), antimicrobial and diagnostic stewardship, research and development, and education and training form the five strategic objectives of the Global Action Plan (GAP) for containing AMR [9]. 

It is well known that prompt use of the correct antibiotic, at the appropriate dose and for the appropriate duration, improves patient outcomes, resulting in decreased mortality and morbidity [10]. Inappropriate use of antibiotics, however, can lead to serious antibiotic-associated adverse drug events (ADEs). *Clostridium difficile* infection (CDI) is widely described and is a serious ADE following the administration of broad-spectrum antibiotics [11]. Furthermore, unlike with other medications, exposure to antibiotics has the potential to induce resistance, making the same drugs ineffective even in cases of no previous exposure [7,12,13]. Appropriate antibiotic use is, therefore, critical. Evidence suggests that antimicrobial stewardship (AMS) is a valuable tool to facilitate rational antibiotic use, reduce healthcare costs and decrease resistance, lessen healthcare-associated infections, and improve patient outcomes [7,12,13,14].

South Africa took a bold step towards curbing resistance in 2014 when the Antimicrobial National Strategy Framework (2014–2024) was endorsed by the Minister of Health. This strategy is designed to: (1) strengthen, coordinate and institutionalize interdisciplinary efforts; (2) optimize the surveillance and early detection of antimicrobial resistance; (3) enhance infection prevention and control; and (4) promote the appropriate use of antimicrobials in human and animal health through AMS [2]. Furthermore, the National Department of Health (NDOH) has published guidelines for the implementation of AMS in South Africa in a One Health context as well as guidelines for the prevention and containment of AMR at the hospital level, in line with the Antimicrobial National Strategy Framework [2,5,15,16,17,18,19]. 

Before the widespread rollout of AMS activities within the province, it is imperative to obtain a baseline overview of what structures and activities are currently in place. To achieve this, we conducted a situational analysis of AMR and AMS in the KZN public healthcare sector to: (1) obtain an overview of AMS activities at public-sector hospital-level facilities which had an antimicrobial stewardship committee (AMSC); (2) identify the challenges experienced in AMS implementation; and (3) provide an overview of the resources available for AMS implementation in order to inform and improve future AMS implementation. 

## 2. Results

### 2.1. Pilot Study

Nine out of twenty-three respondents completed the pilot survey, giving an overall response rate of 39%. Based on the survey feedback, it was evident that all respondents agreed that the survey covered the main aspects of AMS. Fifty-six percent (5, N = 9) of the facilities strongly agreed, while thirty-three percent (3, N = 9) agreed that the survey was understandable. Eighty-nine percent (8, N = 9) agreed that the survey was easy to complete. Six (66%) agreed that the instructions accompanying the survey were clear and understandable and the time to complete the survey was adequate. The time to complete the survey was between 15 min and 7 days with a median of 30 min. In the absence of critique on the survey instrument, the original survey, used in the pilot, was disseminated to the remaining KZN public-sector hospitals, and responses from both the pilot and main study were analysed as a single sample set. 

### 2.2. Main Study 

A total of 58 responses, including responses in the pilot phase of the study, were received; however, one response was omitted due to incomplete submission (only the demographic section was answered). Fifty-seven responses (79%) were, therefore, recorded and analysed. The results were stratified by district, hospital type and whether the hospital had an AMSC (Table 1). Partial completion of certain questions resulted in different denominators for the analysis.

### 2.3. Key Support for the AMS Committee 

#### 2.3.1. Leadership Support

Regarding leadership support, 47% (20, N = 43) of the facilities with an established AMSC had a formal written statement from leadership to support AMS efforts at the facility, and 7% (3, N = 43) had budgeted financial support for AMS activities. The latter included salaries, training, and information technology (IT).

#### 2.3.2. Accountability, and Drug and Antimicrobial Expertise

Eighty-four percent of the facilities (36, N = 43) had appointed a lead for the AMSC. Survey responses for drug and antimicrobial expertise showed that 86% (37, N = 43) had chosen a pharmacist leader, whilst only 37% (16, N = 43) had an on-site or off-site clinical microbiologist, and 5% (2, N = 43) had an on-site infectious disease physician. 

#### 2.3.3. Composition of the AMS Committee and Key Support for the AMS Programme

Staff representation on the AMSCs in the various facilities were as follows: management (84%, 36, N = 43), clinicians (98%, 41, N = 42), pharmacy (100%, 43, N = 43), nursing (93%, 40, N = 43), microbiology (36%, 15, N = 42), and IPC (100%, 43, N = 43). The survey results of the departments or staff that collaborated with the ASP were as follows: clinicians (98%, 42, N = 43), pharmacy (88%, 38, N = 43), IPC (98%, 42, N = 43), microbiology (47%, 20, N = 43), and IT (5%, 2, N = 42). 

### 2.4. Actions to Support the AMS Optimal Antimicrobial Use

#### 2.4.1. Policies and Procedures

Sixty-seven percent (29, N = 43) of the facilities with an AMSC had put a policy in place that required prescribers to document, either on the medical record or prescription order for all antibiotics, the following details: dose, duration, and indication. Sixty-five percent (28, N = 43) of the facilities reported having facility-specific treatment recommendations based on national guidelines, the essential medicines list (EML), and/or local pathogen surveillance data to assist in the selection of appropriate antibiotics for common clinical conditions. Sixty-seven percent (29, N = 43) had an official term of reference, and 23 (53%, N = 43) had a dedicated antimicrobial chart. 

#### 2.4.2. Interventions

Thirty-three percent (14, N = 43) of the facilities conducted multidisciplinary ward rounds. Fifty-one percent (22, N = 43) had AMR surveillance reports available and 36% (15, N = 42) reviewed these surveillance reports. Thirty percent (13, N = 43) had microbiologist input on pathogen surveillance data. Those facilities that had microbiologist input on pathogen surveillance data (aOR: 5.12; 95% CI: 4.08–22.02; *p*-value = 0.001) and microbiological investigations prior to the commencement of antibiotics (aOR: 6.73; 95% CI: 1.08–42.01; *p*-value = 0.041) were significantly associated with on- or off-site microbiology support (Table 2). 

Furthermore, respondents that had microbiologist input on pathogen surveillance data (aOR: 43.54; 95% CI: 4.03–147.65; *p*-value = 0.002) were significantly associated with the membership of a microbiologist on the AMSC (Table 3). Respondents that had a representative from microbiology on the AMSC were significantly associated with having and interrogating facility-specific antibiograms (*P* = 0.051 and *P* = 0.036, respectively) (Table 3). 

The interventions that the AMSC were involved in were divided into broad interdepartmental hospital interventions, pharmacist-specific interventions and diagnosis, and infection-specific interventions. 

#### 2.4.3. Broad Interventions

Some of the broad interventions included: microbiological investigations and consultations prior to antibiotic commencement (38%, 16, N = 42); empiric treatment in line with standard treatment guidelines (STGs) (91%, 39, N = 43); documented indication for antibiotics (88%, 37, N = 42); and review of antibiotic usage in conjunction with culture results (76%, 31, N = 41). Others included a change in antibiotic prescription, i.e., the cessation of therapy/de-escalation/substitution or the addition of more agents (79%, 33, N = 42); intravenous to oral switch (75%, 30, N = 40); and hang-time (54%, 21, N = 39) interventions. Thirty-three percent (14, N = 43) of hospitals had a formal procedure for clinicians to review the appropriateness of all antibiotics 48 h following initial prescription orders. 

#### 2.4.4. Pharmacist-Specific Interventions

Pharmacist-specific interventions included: advising a switch from intravenous to oral antibiotics (74%, 31, N = 42); dose adjustments in cases of organ dysfunction (81%, 35, N = 43); adverse drug reactions (ADRs) (88%, 37, N = 42); drug interactions (93%, 38, N = 41); dose optimization (pharmacokinetics/pharmacodynamics) to optimize the treatment of infection caused by bacteria with reduced susceptibility (70%, 30, N = 43); alerts where therapy may be duplicated (44%, 18, N = 41); and time-sensitive stop orders for specific antibiotic prescriptions (29%, 12, N = 41). Fifty-seven percent (24, N = 42) had dedicated pharmacy ward rounds, albeit in selected wards such as the Intensive Care Unit (ICU) (26%, 6, N = 23); neonatal intensive care unit (NICU) (23%, 6, N = 26); and surgical (45%, 17, N = 38), paediatric (42%, 16, N =38), and medical wards (45%, 18, N = 40). Eight hospitals had other types of wards. These hospitals included specialist TB and psychiatry hospitals. Sixty-two percent (24, N = 40) were involved in medication chart reviews in the outpatient department (OPD). It must be noted that there was no ICU and NICU in 19 and 16 facilities, respectively. Four facilities did not have surgical and paediatric wards, and two did not have a medical ward. 

#### 2.4.5. Diagnosis and Infection-Specific Interventions

The following facilities had infection-specific guidelines in place for optimal use of antimicrobials for the following common infections: community-acquired pneumonia (74%, 31, N = 42), urinary tract infections (UTI) (76%, 32, N = 42), skin and soft-tissue infections (76%, 32, N = 42), surgical prophylaxis (71%, 30, N = 42), methicillin-resistant *Staphylococcus aureus* (64%, 27, N = 42), non-CDI antibiotics in newer cases of CDI (51%, 20, N = 39), culture-proven invasive (e.g., bloodstream) infections (50%, 20, N = 40), HIV (83%, 35, N = 42), TB (83%, 35, N = 42), candidiasis (69%, 29, N = 42), and Cryptococcus (81%, 34, N = 42). 

### 2.5. Tracking: Monitoring Antibiotic Prescribing, Use, and Resistance

The results for the tracking and monitoring of antibiotic prescription, use, and resistance are displayed in Table 4.

The results of the univariate logistic regression model (see Table 5) showed that those facilities that produced an antibiogram (aOR: 4.80; 95% CI: 1.25–18.42; *p*-value = 0.022) were significantly associated with microbiology representation on the AMSC.

### 2.6. Reporting of Information to Staff on Improving Antibiotic Use

A total of 74% (31, N = 42) of the facilities shared facility-specific reports on antibiotic use with their prescribers, 24% (10, N = 42) distributed a current antibiogram to the prescribers of the facility, and 66% (27, N = 41) sent their prescribers direct personalized communication about how they could improve their antibiotic prescribing. Respondents from facilities containing an ID physician were significantly associated with having a current antibiogram distributed to prescribers within the facility (*P* = 0.010), and with sending prescribers personalised communication regarding improving prescription (*P* = 0.044) (Table 6). 

### 2.7. Education 

Sixty-three percent (27, N = 43) of the facilities used their stewardship program to provide education to the clinicians and other relevant staff to improve prescribing. In 51% (21, N = 41) of the facilities, members of the AMSC had attended a form of AMS training. 

### 2.8. Comparing Challenges between Facilities with an AMSC vs. Facilities without an AMSC

**Figure 1:**
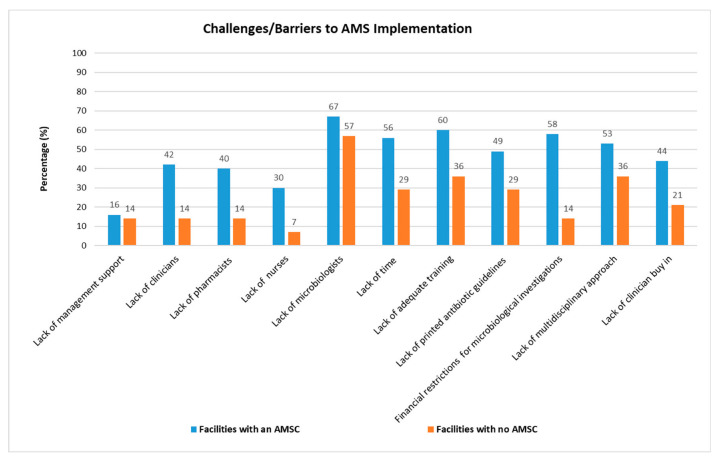
Comparison of challenges between facilities with an antimicrobial stewardship committee (AMSC) vs. facilities without an antimicrobial stewardship committee (AMSC).

Similar challenges or barriers (Figure 1) were identified in facilities that had an AMSC and those that did not. Notably, for all categories of challenges, a higher percentage of challenge was recorded for facilities with an AMSC compared to facilities that did not have an AMSC. 

Other challenges of those with and those without an AMSC are recorded verbatim, as individual comments, in Table 7.

## 3. Discussion 

Since the formation of the Global Antibiotic Resistance Partnership—South Africa (GARP-SA) in 2011, which was later replaced by the South African Antibiotic Stewardship Programme (SAASP), several strategies to combat antimicrobial resistance have been advocated [2,5,15]. South Africa (SA) has a well-established national health governance structure, availability of an EML and STGs, guidelines for antimicrobial prescribing and a national strategy and guidelines for implementation to combat AMR [2,15,20,21,22]. Recently, national guidelines were introduced on the prevention and containment of AMR which include guidance on the implementation of AMS at facility level [5,16]. Consequently, SA falls in the intermediate–advanced stage concerning levels of capacity for core national-level ASP activities and policies, as defined by the CDC for low-and middle-income countries (LMICs) [23]. Therefore we saw it best to adopt and adapt the 2014 seven core standards, as laid out by the CDC for hospital antibiotic stewardship programs, to assess the level of ASP at the public-sector hospital facility level [7]. 

A study by Engler et al., conducted on 26 health facilities including CHCs and hospitals across eight of the nine South African provinces, demonstrated that there was, on average, 59.5% compliance with the National Strategic Framework and implementation guidance for ASPs; the study focused on whether cultures were taken before antibiotic initiation, if a review occurred within 48 hrs of antibiotic prescribing, and if there were audits on surgical antimicrobial prophylaxis [24]. 

A recent study conducted in the Western Cape surveyed the activities of the AMSC in 47 facilities, to ascertain whether facilities had antimicrobial charts; conducted stewardship ward rounds; had an antimicrobial restriction policy in place; conducted audits, training, and antibiogram reviews; promoted awareness; reviewed AMS policy; and had infection prevention and control training [25]. 

To the best of our knowledge, a survey of this magnitude and comprehensiveness on existing AMS activities within the KZN province has not been carried out. To galvanize support, as well as encourage the widespread rollout of AMS activities, it is imperative to obtain a baseline overview of what structures and activities are currently in place. To our knowledge, this is the first published project that has been conducted to determine the type and extent of AMS activities in the KZN public-health sector. 

This situational analysis revealed that at least 75% of public-sector hospital facilities in KZN had set up an AMSC; however, very few AMSCs met regularly. Only one facility had bi-monthly AMS meetings, less than 30% met monthly to discuss AMS, and less than a quarter met every four months. Regular meetings are essential to provide ASP updates as well as identify areas for improvement. It is also a way to provide evidence-based feedback, keep stakeholders motivated, and garner support for future initiatives. If the AMSC is not a standalone body but a subcommittee of the PTC, the AMS role needs to be firmly established alongside more traditional ones of formulary management and patient-safety monitoring [7], with AMS as a standing agenda item at the very least. The survey further revealed that individual ASPs were at various levels of functioning. 

### 3.1. Leadership Support

With less than half of the facilities having a written statement from leadership to endorse AMS initiatives and the absence of a budget for AMS in all but three facilities, it was clear that most facilities did not have adequate leadership and financial support for AMS. 

Leadership commitment is integral to the sustainability and progression of an ASP [7,13]. In SA there is buy-in for AMS from senior management at both national and provincial levels. The survey reveals, however, that there have been challenges in setting up functional AMSCs within the hospitals. Buy-in from hospital senior management would address critical resource allocation such as adequate staff, time, training, program infrastructure, and IT support [7,13,26]. Management endorsement would also elicit support from other healthcare professionals (HCPs) within the facility. Financial support is vital to ASPs [26] and have been shown to improve patient outcomes and reduce antibiotic resistance, making it a worthwhile investment [7,14,23]. ASPs could function under quality assurance and patient safety and should be allocated separate funding [27,28]. Stakeholders should be aware that although there is a cost attached to the setting up of a comprehensive ASP, these programs soon become self-sustainable and have demonstrated considerable cost reductions [7,13,27,29]. In the USA, comprehensive programs at many different hospitals showed a 22–26% reduction in antimicrobial use and an annual cost-saving of $200,000–$900,000 [27]. A recent systematic review looked at the average per-patient cost-saving in the form of bed-day saved due to an ASP. In the EU and UK, the proportion of a bed-day saved represents 60–80% of the cost of a bed day. In the US the proportion was approximately 32% [30]. In KZN, the DOH has taken steps towards budgeted financial support, which includes salaries, training, and information technology (IT). 

Ideally, AMS should be included in job descriptions and performance reviews, with staff time dedicated to AMS activities in their daily routine, as well as participation in and promotion of AMS training and education [7,13]. Some countries (Australia, Belgium, Canada, France, Germany, the Netherlands, Norway, the UK, and the USA) have regulations in place, making hospital stewardship teams mandatory [28]. In 2016, the European Centre for Disease Prevention and Control (ECDC)’s “Proposals for European Union (EU) guidelines on the prudent use of antimicrobials in humans” recommended that staff have salary support and dedicated time to carry out AMS [28,31]. Staffing recommendations for ASPs exist in Australia, Canada, France, Germany, and the Netherlands [28]. SA would benefit from minimum staffing recommendations for ASPs, together with salary support. This requirement has also been included in the recommendations from the global core standards for hospital ASPs [26]. Management should also be made aware that hospital accreditation by the Office of Health Standards Compliance (OHSC) includes AMS as a core assessment criterion. In SA, although there are no separate AMS positions, AMS is being incorporated into existing job descriptions for new pharmacy posts. 

### 3.2. Accountability 

The majority of the facilities surveyed had appointed a leader for the stewardship program. A strong leader takes responsibility for outcomes. Preferably, this should be a physician or pharmacist who has had formal training in infectious diseases or antimicrobial stewardship [7]. In SA, however, evidence suggests that pharmacists or doctors without an infectious disease background can successfully lead an ASP, provided they have had specific AMS training [23,32,33]. A pharmacist-driven AMS program across 47 Netcare hospitals in the private sector reduced antibiotic DDD per 100 patient days from 101.38 (95% CI: 93.05–109.72) to 83.04 (74.87–91.22) in the post-implementation phase (*P* < 0.0001) [32]. In a rural hospital in George, an ASP was developed through outreach from the Groote Schuur Hospital (Western Cape, South Africa). Leadership was allocated on a 6-monthly rotational basis to either a dedicated young doctor or another healthcare professional [33]. 

### 3.3. Drug and Antimicrobial Expertise

Most of the facilities had appointed a pharmacist leader. Clinical microbiologists and infectious disease specialists, however, were identified as scarce human resources. Only about a third had access to clinical microbiology support, and only two facilities had an on-site infectious disease physician. 

Appointing a pharmacist as a co-leader is often recommended, as this provides drug expertise for the ASP [7]. Clinical microbiologists are integral to the success of stewardship. They provide essential diagnostic services, information on biomarkers, rapid diagnostics, antimicrobial susceptibility testing, the testing of new drugs against appropriate pathogens, surveillance, cumulative antimicrobial susceptibility reports (antibiograms), and the provision of education [34]. Due to the relatively low number of specialist clinical microbiologists in KZN in relation to the number of public healthcare facilities in the province, on-site and off-site support is offered. In KZN, there are currently 12 clinical microbiologists. Each specialist has an on-site facility at which they are based, with additional support provided to other facilities within the province which may be located in other districts within KZN. Off-site support refers to the availability for daily telephonic consultation, outbreak management, scheduled off-site visits to the relevant facility, and serving as a member on the AMS committee, should there be one established. 

ID physicians are the recommended leaders for ASPs because they have the ideal training, expertise, and experience [7,27,35]. More often than not, they can influence prescribing patterns; moreover, they can guide diagnostic testing and the interpretation of results. ID physicians are also involved in hospital epidemiology, quality improvement, infection prevention, and patient-safety activities [35]. ID specialists are, however, a scarce human resource in SA. They are a seemingly scarce resource even in the US. In one rural facility, an innovative strategy was to implement a once-a-week remote teleconference with an ID physician wherein cases were reviewed. The ID physician also provided telephonic support to on-site clinicians and assisted with AMS education [36]. A similar approach is used in KZN, wherein the infectious disease specialist provides both on-site and telephonic support within the district in which they are based. 

### 3.4. Composition of the AMS Committee and Key Support for the AMS Programme

Most of the AMSCs had representation from management, clinicians, pharmacy, nursing, and IPC; however, only about a third had a representative from microbiology. 

In SA, there is a two-layered structure for the AMSC. The AMSC comprises the hospital CEO, senior physician or clinical director, assistant manager of pharmaceutical services, nursing service manager, and IPC practitioner [16]. Directly under the AMSC is an AMS team consisting of at least two members responsible for operational activities. The AMSC oversees the coordination of AMS activities and provides six-monthly reports to the provincial AMS committees [16]. Many South African healthcare institutions have learned to adapt to various roles due to resource constraints. Ideally, however, the team should contain the correct mix of expertise, or else AMS initiatives and established prescribing and dispensing policies in the country will not be met. At the bare minimum, it is recommended that AMS teams should include the following key core members: an infectious disease specialist, a pharmacist with infectious disease training, a clinical microbiologist, and an infection control specialist [7,27]. In the US, larger facilities have achieved better AMS success by employing staff dedicated solely to the development and management of AMS activities [7,13,36]. 

Collaboration between IPC and AMS is essential for reducing the development and transmission of resistance [27]. At a busy regional hospital in Durban, a multidisciplinary-team approach consisting of a clinical microbiologist, pharmacist, IPC, and clinicians demonstrated that such a collaboration is possible. The programme is still in its infancy, but to date, 138 patients have been followed up [37]. In SA hospitals, where inter-professional collaboration took place, it was found that this augmented and complemented the ASP [29,37,38]. Perhaps hospitals should look into moving towards an integrated stewardship model combining antimicrobial treatment, infection prevention, and diagnostics (AID) as is used in some hospitals in Germany and the Netherlands. This will optimize patient care whilst minimizing infection spread [39]. It will also allow for greater dissemination of knowledge amongst the different stakeholders, as well as elicit timely diagnostics, treatment plans, and infection prevention [39]. 

#### Key Support for the AMS Program

The involvement and support of key groups within the hospital are integral to the success of any ASP [7]. The results revealed that the vast majority of the facilities had support from clinicians, pharmacists, and IPC professionals. Although remote support is provided in facilities with no on-site clinical microbiologists, less than 50% listed that they had support from microbiology. A mere two facilities had IT support. IT support and infrastructure is currently being rolled out to all facilities at all levels of care in the province. In KZN, the start of the electronic dispensing rollout began in 2019.

The success of an ASP depends largely on the collaboration and participation of all healthcare professionals. Institution-wide uptake of AMS interventions at various facilities in the public and private sectors in SA largely contributed to individual ASP success [29,32,33,37,38]. As the primary prescribers of antibiotics, clinician buy-in significantly aids efforts to improve antibiotic use. Clinicians and departmental heads can also make valuable contributions to policies and interventions [7,13]. Clinical microbiologists are vital in the reporting of resistance and CDI cases [7,13]. Laboratory staff are essential in the diagnostics, creation, and interpretation of antibiograms, which can greatly assist the AMSC [7,13]. Nurses are integral to IPC, making sure samples are sent to the laboratories before antibiotic commencement [7,13], monitoring patients, and alerting clinicians to early signs of infection [40]. Nurses are also well placed to support AMS interventions such as hang times [41], monitoring patients, alerting prescribers and pharmacists with respect to excessive antibiotic durations [40], and administering antibiotics as per the dosing regimen. 

The results clearly show that IT and IT staff are underutilized resources in AMS. IT can be used as a crucial information source (e.g., availability of protocols, facility-specific protocols at the point of prescribing, patient medication records, and monitoring antibiotic use). IT resources also aid in the collection and reporting of data for decision making, e.g., informing rational antibiotic prescribing and formulating guidelines [7,13]. IT-assisted signalling can be used to signal areas for priority action. Budgeting, investment, and integration should be made with regard to IT. Electronic health records (EHCs) with a clinical decision support system would greatly assist the ASP [26]. 

### 3.5. Actions to Support Optimal Antimicrobial Use

#### 3.5.1. Policies and Procedures

Institutions should have national and facility-specific policies and procedures in place to support appropriate antimicrobial use [7]. The majority had official terms of reference and a policy in place for the documentation of dose, duration, and indication; note that around two-thirds consulted national guidelines, the EML, and local pathogen surveillance data. 

Pathogen surveillance data are provided by the on-site and off-site Clinical Microbiologist supporting the facility, to assist clinicians and IPC practitioners with surveillance and antibiotic prescribing. Laboratory surveillance is collated by the National Institute of Communicable Diseases (NICD) from results available from various facilities; this enables the generation of AMR data from different geographical regions in SA. The NICD also monitors and receives resistant isolates from selected hospitals in SA, which are published in a monthly communique. Sentinel surveillance, which involves the submission of pathogens/isolates from selected sites, may not necessarily be representative of all facilities; however, the NICD also performs enhanced and electronic surveillance of AMR, which is compiled annually [42].

It is important to ensure that prescribers abide by the EML and STG recommendations and understand where to access and source national policies and procedures. Local pathogen surveillance data can further assist in antibiotic optimisation for common clinical conditions [7]. A little over half of the facilities had a dedicated antibiotic chart. Boyles et al. found that when charts were combined with antibiotic ward rounds, it reduced antibiotic consumption and pharmacy costs [38]. Documentation of dose, duration, and indication can greatly assist in antibiotic-use evaluation [7]. 

#### 3.5.2. AMS Interventions 

Only about a third of the facilities were involved in multidisciplinary AMS ward rounds, and just over half had AMR surveillance reports. A little over a third reviewed those surveillance reports, whilst less than a third reported that they had a microbiologist input on pathogen surveillance data. Our study demonstrated that respondents that had a representative from microbiology were significantly associated to have and review facility-specific antibiograms. Those facilities that either had on- or off-site microbiology support, or a microbiologist on the AMSC, were significantly associated with having microbiologist input into pathogen-specific data. These facilities were also significantly associated with conducting microbiological investigations prior to the initiation of antibiotics. These results further highlight the importance of having regular access to microbiology support. 

Evidence advocates for multidisciplinary ward rounds for the improvement of antimicrobial use and patient outcomes [7,33,37,38]. A recent prospective study at an academic hospital in the USA revealed that de-escalation is most likely to occur if pharmacists participated in multidisciplinary ward rounds. De-escalations were recommended for 35/39 patients when clinical internal medicine pharmacists joined ward rounds, compared to 13/25 patients on services without pharmacists (*P* = 0.001) [43]. In SA, evidence suggests that multidisciplinary ward rounds are an integral component of stewardship. Boyles et al. demonstrated that ward rounds, together with AMS education and a dedicated antibiotic prescription chart, produced a cost saving of ZAR 3.2 million (USD 208,934) over 4 years [29,38]. Regular microbiology surveillance and empiric antibiotic guidelines have been shown to reduce broad-spectrum antibiotic usage [44]. 

Interventions, however, need to be balanced between the needs of the facility and the capacity of the facility to carry out those interventions. This largely depends on the resources and expertise available [7,23]. 

#### 3.5.3. Broad Interventions

Most of the facilities reported that they initiated empiric treatment in line with STGs, and that the indication for the antibiotic was documented. In facilities adopting broad interventions, the majority reviewed antibiotic usage following the receipt of culture results, and practised a switch from intravenous to oral antibiotics. There were areas, however, that required further interventions. Few facilities asked for microbiological investigations prior to antibiotic initiation, nor did they have a formal procedure in place for clinicians to review antibiotic use 48 h post-initiation, and less than half the facilities were involved in hang-time interventions. 

The majority of antibiotics are commenced empirically. A reassessment of the patient’s needs should be performed once culture results and other diagnostic information become available. Antibiotic “time outs” or antibiotic reviews after 48 h would promote the appropriate use of antimicrobials. Furthermore, they often answer crucial questions such as: Is the infection responding to the antibiotic prescribed? Is the patient on the correct antibiotic? Are dose and route-of-administration adjustments necessary? Is there a need for de-escalation and a review of antibiotic duration [7]? Hang-time, or the time from when the prescription is written to it being administered, influences mortality [41]. For patients in septic shock or severe sepsis, a delay of even one hour could increase mortality by 7.6% [41,45]. Messina et al. carried out a prospective audit across 33 Netcare hospitals, where a hang-time intervention consisting of an educational poster and guide reduced hang times in 78.4% of the patients who received antibiotics [41]. 

#### 3.5.4. Pharmacist-Specific Interventions

Dedicated pharmacy ward rounds were carried out in just over half of the facilities. For most, however, these were only performed on selected wards. This shows a need for more pharmacist involvement at the ward level. About two-thirds of the facilities were involved in AMS chart reviews in the OPD. Pharmacists were fully involved in traditional clinical roles, such as dose adjustments in the case of organ dysfunction, ADRs, drug interactions, IV to oral switch, and dose optimization. The survey results highlight areas where pharmacists could add more value to AMS, such as alerting clinicians on duplicate or unnecessary antibiotic cover, and automatic time-sensitive stop orders (e.g., antibiotics prescribed for surgical prophylaxis). 

Pharmacy interventions during chart reviews have been shown to positively impact antimicrobial usage and expenditure [27]. In examples wherein pharmacists were involved in ward rounds in SA, their participation enhanced the ASP by monitoring for overlapping antimicrobial cover, polypharmacy, dosages, incorrect treatment durations, and irrational prescribing [32,37]. In one program, the pharmacist maintained a database for patient monitoring [37]. A second study reported that 1 in 15 scripts benefitted from pharmacist intervention [32]. 

It can be argued that interventions at the ward level are difficult to achieve if pharmacists, due to resource constraints, are confined to a dispensary and, therefore, do not have access to all the pertinent patient information to make an informed decision. The OPD, however, presents an easy opportunity for pharmacists to become more involved in AMS, especially in an overburdened health system wherein staff shortages often prevent pharmacists from being more involved at the ward level. In the US, the OPD accounts for approximately 60% of the antibiotic expenditure budget. Evidence shows that a 10% reduction in antimicrobial prescribingcould reduce community-associated *C. difficile* infections by as much as 17% [46]. 

#### 3.5.5. Diagnosis and Infection-Specific Interventions

In general, the STG was consulted for the diagnosis and treatment of most common infectious diseases. Guidelines, however, were reported as poorly consulted for methicillin-resistant *Staphylococcus aureu***s** (MRSA), non-*C. difficile* infection antibiotics in new cases of CDI and candidiasis, and culture-proven invasive infections. 

A survey amongst doctors, pharmacists, microbiologists, and nurses in 53 LMICs, revealed that the availability of guidelines combined with education were two of the most effective AMS strategies [47]. In addition to the EML and STG, it is imperative to have facility-specific guidelines and interventions in place. These resources are available in South Africa. Healthcare professionals should be made aware of the availability of guidelines and encouraged to use the evidence in addressing AMR. Guidelines and interventions are important in optimizing therapy, ensuring that the correct diagnostic tests are carried out, and evaluating the antibiotic(s) used for defined infections [7,13]. Diagnostic stewardship is a vital component of appropriate antibiotic selection and infection prevention. Efforts should be made to increase the capacity for diagnostic testing within the facility [23,48]. 

### 3.6. Tracking: Monitoring Antibiotic Prescribing, Use, and Resistance

#### 3.6.1. Process Measures

Slightly more than 50% of the facilities were involved in the monitoring of adherence to policy documentation and monitored compliance with one or more AMS interventions that the hospital had in place. Less than half, however, monitored facility-specific treatment recommendations.

The evaluation of ASP initiatives, e.g., adherence to policies and procedures, is crucial to assess the impact of interventions, and also identify gaps for improvement [23]. Several studies have been carried out in SA using prescription audits to evaluate antibiotic usage against treatment guidelines [49,50,51]. Similar audits can be initiated on a small scale in dedicated areas, e.g., specialised wards such as the ICU, and expanded to larger scale reviews to include all wards, with feedback to empower and educate all health care professionals.

#### 3.6.2. Antibiotic Use and Outcome

This was an area in which more facilities could become involved. Less than 40% of the facilities tracked CDI infections and less than half produced an antibiogram. These interventions, however, require input from microbiology, which was identified as a scarce human resource. All aspects of antibiotic use and consumption were poorly monitored. The indicator that was most frequently used was direct expenditure or purchasing costs.

Clinical outcomes such as a reduction in CDI and resistance can be important indicators of the impact of the AMS interventions in improving antibiotic use [7]. A study relating the effect of the reduction of high-risk antibiotic (HRA) prescribingon CDI rates demonstrated that in one speciality—medicine—a relative 33% (95% CI: 11–56) reduction in HRA led to an estimated reduction in CDI of seven cases/1000 admissions (relative change—24% (95% CI: −55 to 6)) [52].

Antibiotic use and consumption are also important measures of interventions. The Anatomical Therapeutic Chemical (ATC) classification and Defined Daily Dose (DDD) are the gold standards with regard to drug-utilization monitoring and research [53]. In two South African studies, the use of DDDs as a measure of antibiotic use served as an indicator that the program was working [29,32,38]. The global point-prevalence survey (global PPS) was able to collect data on antimicrobial use and consumption from 303 hospitals in 53 countries using an online tool. These countries also included LMIC and upper–middle-income countries. It looked at antimicrobials that are currently under surveillance according to the WHO ATC classification [54]. The global PPS tool can be used to map antimicrobial prescribing and resistance. It provides an easy-to-use tool to complement the WHO Antimicrobial Resistance Surveillance system [54,55]. Guidance on measuring antimicrobial use and consumption and point prevalence studies are available from the WHO [53]. Antibiotic stewardship leading to appropriate consumption of antimicrobials can promote cost reduction and, therefore, serve as a motivator for management as a cost-saving example [7]. Monitoring of an ASP is often difficult because of the lack of access to electronic health-records and centralized prescription-databases [23]. In the private sector, the availability of these records makes prescription audits easier. More effort needs to be dedicated to the implementation and use of electronic health-records in public-sector hospitals. 

#### 3.6.3. Reporting Information to Staff on Improving Antibiotic Use and Education

The majority of the facilities shared facility-specific reports on antibiotic use or communicated with their prescribers on how to improve antibioticprescribing. The current antibiogram distribution to prescribers within the facility was poorly reported. Just under two-thirds of the facilities provided AMS education to relevant staff, and in slightly more than half, members of the AMSC had AMS training. It was not surprising that respondents who had microbiology representation on the AMSC were significantly associated with producing more antibiograms, and respondents from facilities containing an ID physician were significantly associated with having a current antibiogram distributed to prescribers within the facility, as well as providing personalized communication regarding improving prescribing. 

A prospective audit and feedback study conducted by a clinical pharmacist and ID physician in the US demonstrated a 22% decrease in the prescribing of parenteral broad-spectrum antibiotics over 7 years. There was also an accompanying reduction in CDI and nosocomial infection caused by drug-resistant *Enterobacterales* [27,56]. The ASP should serve as a vehicle to provide the facility with updates on antibiotic prescribing, use, consumption, and resistance, as well as infectious disease management [7]. In a recent scoping review of AMS in SA, we found that those AMS initiatives which were accompanied by education and ongoing audits thrived and were sustainable [20]. 

Several studies in LMICs amongst healthcare students and professionals have identified gaps in knowledge, attitudes, and practices (KAP) on AMR and AMS [23,57,58,59,60,61,62,63]. The need for more coordinated and standardized AMS education at undergraduate and in-service levels was highlighted in a recent scoping review [20]. Although KZN lacks a dedicated AMS educational centre, the province has a Regional Training Centre which falls under Human Resource Development. This is a directorate that identifies and facilitates training needs with other directorates within the country. There are also various online courses which are available covering general AMS principles [64,65]. AMSCs should encourage staff participation in AMS education. Policies and disease-specific guidelines are in place, but the existing data show that often, HCPs require a refresher and in-service training around these guidelines to maintain competence. Repetitive ongoing training often re-enforces principles and can greatly enhance a program [23]. 

### 3.7. Challenges Experienced in the Implementation of AMS Activities

A comparison of the challenges of those with an AMSC and those without revealed that both had similar challenges/barriers to AMS implementation. These included: suboptimal management support; a lack of human resources, time, training, and printed antibiotic guidelines; the lack of a multidisciplinary approach; low clinician buy-in; no access to a microbiologist; and financial restrictions regarding microbiological investigations. What was surprising was that the percentage for the different challenges in the group with an AMSC was higher than those without. Individual facilities also voiced the need for specific DOH antimicrobial guidelines, citing limitations to the EML, drug availability, leaving the responsibility for AMS solely to the pharmacy department, and a lack of strong leadership. Others expressed concern that there were no dedicated AMS meetings as the discussion took place at PTC meetings, and that some stakeholders did not have time because of competing work priorities, which also required meetings. 

SA has several infectious disease guidelines that are regularly updated. Certain prescription guidelines for antibiotics, however, are not regularly updated for the province, which can be challenging. For the majority of the facilities serviced by a clinical microbiologist in the public sector, local (facility) antibiotic prescribing guidelines are compiled based on surveillance data and consultation between clinicians and the clinical microbiologist. The guidelines within the facility are reviewed and updated yearly based on pathogen surveillance and susceptibility profiles. It was not surprising that the challenges cited in this study are similar to those experienced in LMICs. These include: inadequate infection-specific guidelines and AMS training, diagnostic and laboratory ability, human, time, and financial resources [23,26,28,47]. Limited access to appropriate antimicrobials can also increase inappropriate antibiotic use and resistance [23,47]. Other challenges that have been cited by LMICs include lack of expertise, hospital leadership, and commitment; inadequate collaboration; an absence of prescriber support; minimal commitment from nurses; and suboptimal use of IT [26].

Possible solutions/strategies to some of these challenges have been laid out by the Global Core Standards for hospital antimicrobial stewardship programs [26]. A lack of expertise can be overcome by using a “train the trainer” model to build capacity. In 2018, DOH facilitated a comprehensive interprofessional two-and-a-half-day AMS workshop, targeting pharmacists and doctors at an operational level. This was led by Sefako Makgatho Health Sciences University. This training was positively received, prompting some KZN Districts, e.g., Umzinyathi and Ugu, to engage with Provincial AMSCs to assist with the facilitation of future District AMS training. 

Hospital leadership could build stewardship functions into job descriptions, and stewardship outcomes could be incorporated into key performance indicators for hospitals [26]. Each hospital should look into an ASP financing model. Considering the scale of AMR, national or regional funding sources could be considered. Collaboration between all healthcare professionals could be increased by appointing a multidisciplinary AMS team, auditing ASP initiatives, and providing feedback. Involving nurse leaders in ASP decision making would elicit greater nurse participation. The provision of tailored communication and nurse-focussed training in stewardship often further assists in encouraging nurse support [26].

Overall, however, the results were encouraging, in that they demonstrated that despite facing numerous challenges, the majority of the facilities had still set up an AMSC, and some were actively engaged in AMS interventions. 

## 4. Limitations 

We only collected detailed information on AMS activities if the facility responded to having a formal AMSC, i.e., hospitals without an AMSC could still be conducting activities. Similarly, there is a likelihood that a response bias existed, in that those facilities that had a formal AMSC might have been more likely to respond to the survey vs. those facilities without an AMSC. 

The findings reported in this paper are based on a 79% response rate. Fifteen facilities did not respond; therefore a composite view of AMS activities at all facilities in the province could not be compiled. This was a survey recording the compliance to the seven core CDC standards, which gave a surface view of what is happening at the facility level [7]. This study served to quantify whether something was in place or not. Further studies, however, will need to be conducted to extract a more in-depth insight into the quality of AMS activities at individual facilities and at different levels of care.

## 5. Conclusions

The survey indicates that although most public-sector hospital facilities in KZN have set up an AMSC, individual ASPs are at various levels of functioning. For the majority, basic structures are in place to conduct AMS activities. To scale up AMS activities to a more advanced level, however, requires the deployment of additional support and resources (financial, staff, time, IT, and education). There was suboptimal IT use, and specialists in microbiology and infectious diseases were identified as scarce human resources. More frequent AMS meetings are required for the ongoing assessment of current ASPs and to identify future needs. There is also a need for more pharmacy involvement in ward rounds. Despite the challenges and barriers of suboptimal levels of staff, time, finance, and other resources, the results show that many AMS initiatives can and have been implemented in some KZN public-sector hospitals. These ASPs were, however, in the early implementation phase, and there is a need for further improvement. 

## 6. Materials and Methods 

### 6.1. Ethical Considerations

Department of Health (DOH) permission, gate-keeper access, and ethical approval from the Biomedical Research and Ethics Committee (BREC) (BE560/18) were obtained before the commencement of the study. 

### 6.2. Study Setting 

**Figure 2:**
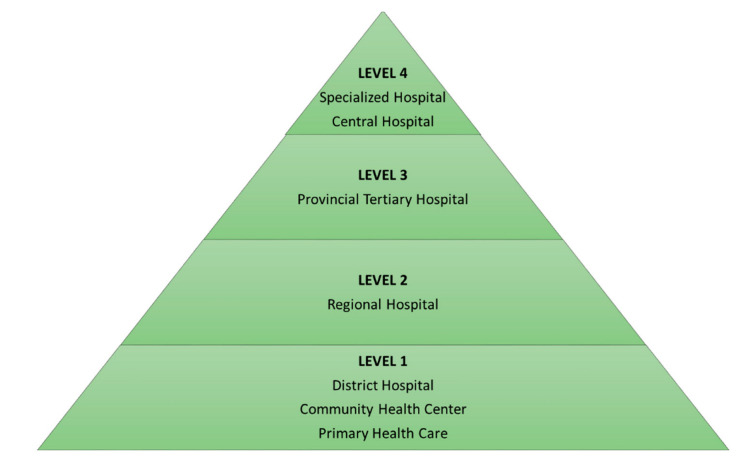
Levels of healthcare in South Africa [66].

The public healthcare sector comprises 5 categories of hospitals, viz., district, regional, tertiary, central, and specialized, with each category providing different levels of care (Figure 2) [66,67]. The district hospitals receive referrals from community healthcare centres (CHCs) and primary healthcare (PHC) clinics, and provide services such as diagnostics, treatment, care, and counselling. Patients who require management at a higher level of care are referred to a regional hospital, which provides specialist support to several district hospitals. The provincial tertiary hospital accepts referrals and provides sub-specialist support to regional hospitals by offering specialised services for all disciplines. Patients who require management at a higher level of care are referred to a central hospital, which has very highly specialized units and provides multi-speciality clinical services and innovation. Specialized hospitals provide management for specialities such as psychiatry and multi-drug-resistant tuberculosis (MDR-TB) [66]. 

In KZN, there are 43 district hospitals, 10 regional hospitals, 3 tertiary hospitals, 1 central hospital, and 15 specialised hospitals.

The survey was conducted at all public-sector tertiary, regional, district, and specialized hospitals in KZN, as listed on the KZN DOH website (http://www.kznhealth.gov.za/hospitals.htm, accessed 10 September 2018) [68].

### 6.3. Study Design

This was a cross-sectional, quantitative study. A survey (Supplementary S2), endorsed by the KZN DOH, was developed in accordance with the CDC [7] and KZN Department of Health Survey Instruments (Supplementary File S1). The survey was electronically disseminated via email through the KZN Pharmaceutical Services and the KZN DOH intranet. 

### 6.4. Data-Collection Instrument 

In this situational analysis questionnaire (Supplementary, File S2), we combined and adapted the CDC checklist associated with its “Core Elements of Hospital Antibiotic Stewardship Programs” [7] and Department of Health Survey Instruments (Supplementary File S1), to create a single tool to investigate the KZN hospitals’ AMS needs in a South African health context. The survey encompassed demographic information (name of hospital, district, name and designation of the respondent, type and number of staff members, number of beds, hospital type, and whether there was a functional AMS Committee (AMSC) at the facility); it also encompassed the seven core elements of AMS, i.e., (1) leadership support, (2) accountability, (3) drug expertise, (4) actions to support optimal antibiotic use, (5) tracking (monitoring antibiotic prescribing, use, and resistance), (6) reporting information to staff on improving antibiotic use, and (7) resistance and education, as well as challenges/barriers to AMS implementation [7]. 

### 6.5. Sample Sites 

#### Pilot Survey 

The survey was initially piloted from December 2018 to April 2019 in 22 randomly selected facilities, stratified by level of care, in each district. Two facilities per district were randomly selected using Microsoft Excel^®^. The 23rd site was conveniently selected from the list of 72 sites. This hospital was known to have an established in-house ASP and was included to provide insight into the completeness of the survey. Feedback on the appropriateness and completeness of the survey concerning AMS was then elicited from the pilot facilities via a Likert-type survey (Supplementary, File S3). 

In the absence of critique on the survey instrument, the unchanged questionnaire was then disseminated to the remaining public sector KZN hospitals (July–December 2019, February–March 2020). 

### 6.6. Data Analysis 

Answers to closed- and open-ended questions were double-checked, captured and coded in Microsoft Excel^®^, then exported to Statistical Package for the Social Sciences (SPSS), version 25, for data analysis. The denominator was the number of hospitals that responded to a question. The numerator was the number of “Yes” responses. The “Yes” responses were recorded as affirmative of what was in place at the facility, unless they stated otherwise under the comments. Non-responses were excluded. Partial completion of certain questions resulted in different denominators for the analysis. Descriptive statistics were performed using Microsoft Excel^®^. Categorical data were described as frequency and percentages. Chi square/fisher exact test was used to assess the bivariate association between categorical variables of interest.

Only statistically significant results are displayed in the manuscript. To view the full results, please refer to the supplementary paper (Supplementary Tables S1–S4). Additionally, univariate and multivariate logistic regression were used to determine the effects of covariates. All the tests were two-tailed, and the criterion for statistical significance was set at a 5% level. 

In the “other challenges” section that the respondents in facilities both with and without an established AMSC had completed, text responses were recorded verbatim. Text responses were analysed using manifest analysis and coding. The responses were condensed to meaning units and codes, and finally, grouped into themes. 

## Figures and Tables

**Table 1:** Responses stratified by district, hospital type, and existing antimicrobial stewardship committee (AMSC).

Study Setting	Number	Percentage
***District***		
Amajuba	3	5%
Ethekwini	16	28%
Harry Gwala	6	11%
Ilembe	4	7%
King Cetshwayo	3	5%
Ugu	2	3%
Umgungundlovu	9	16%
Umkhanyakude	4	7%
Umzinyathi	4	7%
Uthukela	2	4%
Zululand	4	7%
***Hospital Type***		
District	30	53%
Regional	10	17%
Tertiary	3	5%
Central	1	2%
Specialized	12	21%
District and Specialized TB	1	2%
***Specialized Hospital***	12	21%
Psychiatry	4	7%
TB	5	9%
Chronic Rehabilitation	2	3.50%
Ophthalmology	1	2%
***Facilities that had an antimicrobial stewardship committee (AMSC)***	43	75%
***Antimicrobial stewardship (AMS) Meetings***	**N = 43**	
Bi-monthly	1	2%
Monthly	12	28%
Quarterly	8	19%
*Quarterly AMS combined with PTC meetings*	2	5%

**Table 2:** Bivariate Chi-squared test of association and multivariable logistic regression: associations between drug and antimicrobial expertise (clinical microbiologist) and different AMS interventions.

Interventions	Drug and Antimicrobial Expertise, n (%)			aOR (95% CI)	*p*-Value
	Is There a Clinical Microbiologist on-Site or Is There off-Site Support from a Clinical Microbiologist?		Chi-Square *p*-Value		
	Yes	No			
**Microbiologist input on pathogen surveillance data**					
Yes	11 (84.6)	2 (15.4)	0.000 **	5.12 (4.08–22.02)	0.001 **
No	5 (16.7)	25 (83.3)		1	
**Microbiological investigations prior to commencement of antibiotics**					
Yes	10 (62.5)	6 (37.5)	0.011 *	6.73 (1.08–42.01)	0.041 *
No	6 (23.1)	20 (76.9)		1	

Key: aOR—adjusted odds ratio; statistical significance: (*) *P* < 0.05 and (**) *P* < 0.01.

**Table 3:** Bivariate Chi-squared test of association and multivariable logistic regression: associations between composition of the antimicrobial stewardship committee (clinical microbiologist) and different AMS interventions.

Interventions	Composition of the Antimicrobial Stewardship Committee, n (%)			aOR (95% CI)	*p*-Value
	Is there a Representative from Microbiology		Chi-Square *p*-Value		
	Yes	No			
**Availability of AMR surveillance** **reports**					
Yes	12 (54.5%)	10 (45.5%)	0.051 *	3.21 (0.22–46.52)	0.389
No	5 (25%)	15 (75%)		1	
**Interrogation of AMR surveillance reports**					
Yes	9 (60%)	6 (40%)	0.036 *	0.51 (0.03–9.18)	0.647
No	7 (26.9%)	19 (73.1%)		1	
**Microbiologist input on pathogen surveillance data**					
Yes	12 (92.3)	1 (7.7%)	0.000 **	43.54 (4.03–147.65)	0.002
No	5 (17.2%)	24 (82.8%)		1	

Key: aOR—adjusted odds ratio; statistical significance: (*) *P* < 0.05 and (**) *P* < 0.01.

**Table 4:** Tracking: monitoring antibiotic prescribing, use, and resistance.

TRACKING: MONITORING ANTIBIOTIC PRESCRIBING, USE, AND RESISTANCE		
PROCESS MEASURES	N	Responses n (%)
Does your stewardship program monitor adherence to a documentation policy (dose, duration, and indication)?	43	25 (58%)
Does your stewardship program monitor adherence to facility-specific treatment recommendations?	43	19 (44%)
Does your stewardship program monitor compliance with one or more of the specific interventions in place?	42	23 (55%)
**ANTIBIOTIC USE AND OUTCOME MEASURES**		
Does your facility track rates of *C. difficile* infection?	41	14 (34%)
Does your facility produce an antibiogram (cumulative antibiotic susceptibility report?)	42	20 (48%)
**Does your facility monitor antibiotic use (consumption) at the unit and/or facility-wide level by one of the following metrics?**		
By counts of antibiotic(s) administered to patients per day (Days of Therapy; Directly Observed Therapy)?	43	14 (33%)
By number of grams of antibiotics used Defined Daily Dose (DDD),Anatomical Therapeutic Classification)?	43	10 (23%)
By direct expenditure for antibiotics (purchasing costs)?	43	24 (56%)

**Table 5:** Bivariate Chi-squared test of association and univariate logistic regression: associations between composition of the antimicrobial stewardship committee (clinical microbiologist) and tracking: monitoring antibiotic prescribing, use and resistance.

Tracking: Monitoring Antibiotic Prescribing, Use, and Resistance	Composition of the Antimicrobial Stewardship Committee, n (%)			aOR (95% CI)	*p*-Value
	Is There a Representative from Microbiology		Chi-Square *p*-Value		
	Yes	No			
**Does your facility produce an antibiogram**					
Yes	12 (60%)	8 (40%)	0.019 **	4.80 (1.25–18.42)	0.022 *
No	5 (23.8%)	16 (76.2%)		1	

Key: aOR—adjusted odds ratio; statistical significance: (*) *P* < 0.05 and (**) *P* < 0.01.

**Table 6:** Bivariate Chi-squared test of association: associations between drug and antimicrobial expertise (infectious disease physician) and reporting information to staff on improving antibiotic use.

Reporting Information to Staff on Improving Antibiotic Use	Drug and Antimicrobial Expertise, n (%)		
	Is There an Infectious Disease Physician on Site		Chi-Square *p*-Value
	Yes	No	
**Has a current antibiogram been distributed to prescribers at the facility?**			
Yes	2 (20%)	8 (80%)	0.010 **
No	0 (0%)	32 (100%)	
**Do prescribers ever receive direct, personalized communication about how they can improve their antibiotic prescribing?**			
Yes	0 (0%)	27 (100%)	0.044 *
No	2 (14.3%)	12 (85.7%)	

Key: statistical significance: (*) *P* < 0.05 and (**) *P* < 0.01.

**Table 7:** Qualitative results recorded verbatim from survey responses outlining the challenges and barriers to implementation of antimicrobial stewardship (AMS), at facilities with or without an AMS committee, in public-sector hospitals in KZN.

Individual Comments Recorded Verbatim	Themes
*Lack of clear guidelines from DOH regarding antimicrobials.* *Limitations of the EDL*	Limitations in guidelines and EDL
*Lack of drug availability*	Inadequate drug availability
*The AMS Sub Committee is part of the Pharmacy and Therapeutics Committee (PTC), no dedicated AMS committee. Discussion takes place in PTC meetings*	AMS meetings are combined with PTC meetings
*Leadership has not been strong and doctor attendance has been minimal.*	Inadequate strong leadership
*Lack of nursing buy-in*	Inadequate nursing support
*Not the appropriate expertise available to have a fully functional AMS committee.*	Inadequate expertise
*Challenge has been time to have all members for a meeting to draft terms of reference due to clashing responsibilities.*	Inadequate time
*Stakeholders do not have the time due to many other facility meetings held daily. AMS is a standing item on the PTC and IPC agenda. There is no standalone AMS committee*	Competing responsibilities
*The AMS program exists in terms of what the pharmacy staff can contribute.*	The responsibility lies with pharmacists
*An AMS program with guidelines was designed and presented by the clinical pharmacist to the PTC with very underwhelming response. Although we do not have a functional AMS* *program, the clinical pharmacist does carry out some of the pharmacy-related activities*	Suboptimal PTC buy-in

## Supplementary Materials

The following supporting information can be downloaded at: https://www.mdpi.com/article/10.3390/antibiotics11070881/s1, File S1: Situational Analysis: Antimicrobial Stewardship (AMS) Committees at KwaZulu-Natal (KZN) District Level; File S2: Situational Analysis: Antimicrobial Stewardship (AMS) Committees at KwaZulu-Natal (KZN) Hospitals; File S3: Feedback on Antimicrobial Stewardship (AMS) Survey; Table S1: Bivariate Chi-squared test of association and Multivariable logistic regression: Associations between Drug and Antimicrobial Expertise (clinical microbiologist)/Drug and Antimicrobial Expertise (Infectious disease physician)/Composition of the Antimicrobial Stewardship (clinical microbiologist) and different AMS Interventions; Table S2: Bivariate Chi-squared test of association and Univariate logistic regression: Associations between Drug and Antimicrobial Expertise (clinical microbiologist)/ Composition of the Antimicrobial Stewardship (clinical microbiologist) and Tracking: monitoring antibiotic, prescribing, use, resistance and Bivariate Chi-squared test of association and Multivariable logistic regression: Associations between Drug and Antimicrobial Expertise (Infectious disease physician and Tracking: monitoring antibiotic, prescribing, use, resistance; Table S3: Bivariate Chi-squared test of association and Univariate logistic regression: Association between composition of the Antimicrobial Stewardship (clinical microbiologist) and distribution of current antibiogram. Bivariate Chi-squared test of association and Multivariable logistic regression: Associations between Drug and Antimicrobial Expertise (Infectious disease physician); Table S4: Bivariate Chi-squared test of association and Multivariable logistic regression: Associations between Drug and Antimicrobial Expertise (Pharmacist Leader) and Interventions, Tracking (Monitoring antibiotic prescribing, use and resistance) and reporting information to staff on improving antibiotic use.
