# Supplementary material for: Antimicrobial Stewardship in Public-Sector Hospitals in KwaZulu-Natal, South Africa
Source: Antibiotics (Basel). 2022 Jun 30;11(7):881. doi: 10.3390/antibiotics11070881 (PMC9311864; doi:10.3390/antibiotics11070881)
Supplement: Supplementary file 1 [file antibiotics-11-00881-s001.zip › antibiotics-1751082-supplementary.pdf]

Antimicrobial Stewardship in Public Sector Hospitals in KwaZulu-Natal, South Africa

Sarentha Chetty[1,2*], Millidhashni Reddy[3], Yogandree Ramsamy[4], Vusi C. Dlamini[5], Rahendhree Reddy-Naidoo[5], Sabiha Y. Essack[6]

[1]Department of Pharmacy and Pharmacology, Faculty of Health Sciences, University of the Witwatersrand, Johannesburg, South Africa

[2]Discipline of Pharmaceutical Sciences, School of Health Sciences, Westville Campus, University of KwaZulu-Natal, Durban, South Africa

[3]Essential Medicine Consulting, Durban, South Africa
[4]Department of Medical Microbiology, Prince Mshiyeni Memorial Hospital - National Health Laboratory Services, Antimicrobial Research Unit, University of Kwazulu-Natal, Durban, South Africa
[5] Pharmaceutical Services, Department of Health, Kwazulu-Natal, Durban, South Africa,
[6]Antimicrobial Research Unit, College of Health Sciences, University of KwaZulu-Natal, Durban, South Africa

S 1

Situational Analysis: Antimicrobial Stewardship (AMS) Committees at KwaZulu-Natal (KZN) District Level

The Minister of Health launched the National Antimicrobial Resistance (AMR) Strategy Framework on the 16th October 2014. The strategy outlines the country's framework for managing antimicrobial resistance to limit further increases in resistant microbial infections. By ensuring the appropriate prescribing and use of antimicrobials in South Africa, the efficacy of antibiotics can be conserved for the optimal management of infections in human and animal health, and improve patient outcomes.

The KZN AMS Task Team appointed by the Head of Health, Kwa-Zulu Natal is conducting a situational analysis to evaluate the status of AMS committees at district level.

The following five questions should be answered for the district level. Please complete the questionnaire and return via electronic mail to **Pharmacy.Ho@kznhealth.gov.za** by the **Wednesday 24th May 2017**.

1. Name of District……………………………………………………………………………………………………..
2. Name and Designation of Respondent ………………………………………………………………………………
3. Is there a functional Antimicrobial Stewardship committee at the district level?
    a. Yes
    b. No
    c.
4. If Yes, are the following activities conducted by the committee

<table>
<tr><th colspan="2">Activities</th><th>Tick All that Apply</th></tr>
<tr><td colspan="2">Monthly AMS Team Meetings</td><td></td></tr>
<tr><td colspan="2">Multidisciplinary stewardship teams and rounds</td><td></td></tr>
<tr><td rowspan="7">Prospective audits with feedback to prescribers which includes:</td><td>Microbiological investigations prior to commencement of antibiotics</td><td></td></tr>
<tr><td>Empiric treatment against Standard Treatment Guidelines (STGs)</td><td></td></tr>
<tr><td>Documented indication for antibiotics</td><td></td></tr>
<tr><td>Review of antibiotic with culture results</td><td></td></tr>
<tr><td>Change in antibiotic – stopping / de-escalation / substitution / addition of agents</td><td></td></tr>
<tr><td>Intravenous (IV) to oral switch</td><td></td></tr>
<tr><td>Hang time (time from prescription to patient receiving treatment)</td><td></td></tr>
<tr><td colspan="2">Microbiological investigations prior to commencement of antibiotics</td><td></td></tr>
<tr><td colspan="2">Availability and interrogation of AMR reports</td><td></td></tr>
<tr><td colspan="2">Microbiologist input on resistance data</td><td></td></tr>
</table>

Other, Specify:	

5. If No, what are the barriers to the establishment of an active AMS committee?

**Activities**	**Tick All that Apply**
Lack of management support	
Lack of expertise e.g. no microbiologist	
Lack of time	
Lack of training	
Lack of printed antibiotic guidelines	
Financial restrictions regarding microbiological investigations	
Lack of multidisciplinary approach	
Lack of and clinician buy in	
Other, Specify:	

S 2

Situational Analysis: Antimicrobial Stewardship (AMS) Committees at KwaZulu-Natal (KZN) Hospitals

The Minister of Health launched the National Antimicrobial Resistance (AMR) Strategy Framework in 2014. The strategy outlines the country's framework for managing antimicrobial resistance to limit further increases in resistant microbial infections. By ensuring the appropriate prescribing and use of antimicrobials in South Africa, the efficacy of antibiotics can be conserved for the optimal management of infections in human and animal health, in addition to improving patient outcomes.

The KZN AMS Committee (KZN-AMSC) appointed by the Head of Health, Kwa-Zulu Natal is conducting a situational analysis survey to evaluate the status of antimicrobial stewardship (AMS) committees at facility level. The Pharmaceutical Services in KZN and the secretariat of the KZN-AMSC requires assistance from the Chief Executive Officer (CEO) and hospital facility manager in completing the following questions.

Please complete the questionnaire and return via electronic mail to Pharmacy.HO@kznhealth.gov.za. For questions please email Pharmacy.HO@kznhealth.gov.za., chettys4@ukzn.ac.za or call Ms Rahendhree Reddy on 033-8467266.

SECTION A GENERAL INFORMATION

6. Name of District……

7. Name of Hospital……

8. Please circle the level of hospital: a. tertiary
   b. regional
   c. district
   d. specialized

If a specialized hospital please specify?...

9. The number of beds at your facility……………………………………………………………………………….

10. Name and Designation of Respondent ……………………………………………………………………………..

11. Please complete the following details:

The number of:	
a. Clinicians	
b. Pharmacists	
c. Nurses	
d. Microbiologist	
e. Infection prevention and control (IPC) staff	

12. Is there an Antimicrobial Stewardship (AMS) committee at your hospital?

a. Yes
b. No

If yes please complete sections B and C. If No Skip to Section D.

SECTION B KEY SUPPORT FOR THE AMS COMMITTEE

1. LEADERSHIP SUPPORT	Yes	No
A formal written statement of support from leadership to support AMS efforts at the facility		
Budgeted financial support for AMS stewardship activities (Salary, training, information technology (IT)		

2. ACCOUNTABILITY	Yes	No
An appointed physician/pharmacist/nurse lead responsible for stewardship outcomes at your facility		

3. DRUG AND ANTIMICROBIAL EXPERTISE	Yes	No
Is there a pharmacist leader responsible for improving antibiotic use at the facility		
Is there a clinical microbiologist on site or is there off-site support from a clinical microbiologist		
Is there an infectious disease physician on site		

4. COMPOSITION OF THE AMS COMMITTEE	Yes	No
Is there a representative from management		
Is there a representative from clinicians		
Is there a representative from pharmacy		
Is there a representative from nursing		
Is there a representative from microbiology		
Is there a representative from Infection Prevention Control (IPC)		

5. KEY SUPPORT FOR THE AMS PROGRAM<br>*Do any of the departments/staff listed below collaborate with the AMS committee*	Yes	No
Clinicians		
Nursing		
Pharmacy		

Microbiology		
Information Technology (IT)		

SECTION C. ACTIONS TO SUPPORT OPTIMAL ANTIMICROBIAL USE

1. POLICIES AND PROCEDURES	Yes	No
Does your facility have a policy that requires prescribers to document in the medical record or during prescription order entry a dose, duration, and indication for all antibiotic prescriptions?		
Does your facility have facility-specific treatment recommendations, based on national guidelines, Essential Medicines Lists and/or local pathogen surveillance data, to assist with antibiotic selection for common clinical conditions?		
Monthly AMS Team Meetings		
Does the committee have an official "Terms of Reference"		
Does the facility use a dedicated antimicrobial prescription chart		
Other, Specify:		

**2. INTERVENTIONS**<br>*Are the following interventions conducted by the AMS committee*	**Yes**	**No**
Multidisciplinary antimicrobial stewardship ward rounds		
Availability of AMR surveillance reports		
Interrogation of AMR surveillance reports		
Microbiologist input on pathogen surveillance data		
**BROAD INTERVENTIONS**		
Microbiological investigations prior to commencement of antibiotics		
Empiric treatment in line with Standard Treatment Guidelines (STGs)		
Documented indication for antibiotics		
Review of antibiotic with culture results		
Change in antibiotic prescribing – cease therapy / de-escalation / substitution / addition of agents		
Is there a formal procedure for all clinicians to review the appropriateness of all antibiotics 48 hours after the initial prescription orders (e.g. antibiotic time out)?		
Intravenous (IV) to oral switch		
Hang time (time from prescription to patient receiving treatment)		
**PHARMACIST SPECIFIC INTERVENTIONS**		
Are there dedicated pharmacy ward rounds?		
Are chart reviews conducted in the following wards:		
Intensive Care Unit (ICU)		
Neonatal Intensive Care Unit (NICU)		
Surgical		
Paediatric		
Medical wards		
Out Patients Department (OPD)		
Other wards, Please specify		
Advising switch from intravenous to oral antibiotic therapy in appropriate situations?		
Dose adjustments in cases of organ dysfunction?		
Adverse drug reactions		
Drug interactions		
Dose optimization (pharmacokinetics/pharmacodynamics) to optimize the treatment of organisms with reduced susceptibility		
Automatic alerts in situations where therapy might be unnecessarily duplicative?		
Time-sensitive automatic stop orders for specified antibiotic prescriptions?		
Other, Specify:		

**3. DIAGNOSIS AND INFECTION SPECIFIC INTERVENTIONS**<br>*Does your facility have specific guidelines in place to ensure optimal use of antimicrobials to treat the following common infections?*	**Yes**	**No**
Community-acquired pneumonia		
Urinary tract infections		
Skin and soft tissue infections		
Surgical prophylaxis		
Methicillin-resistant Staphylococcus aureus (MRSA)		
Non-C. Difficile infection (CDI) antibiotics in new cases of CDI		
Culture-proven invasive (e.g., blood stream) infections		
HIV (drug sensitive and drug resistant)		
TB (drug sensitive and drug resistant)		
Candidiasis		
Cryptococcus		
Other, Specify:		

**4. TRACKING: MONITORING ANTIBIOTIC PRESCRIBING, USE, AND RESISTANCE**	**Yes**	**No**
**PROCESS MEASURES**		
Does your stewardship program monitor adherence to a documentation policy (dose, duration, and indication)?		
Does your stewardship program monitor adherence to facility-specific treatment recommendations?		
Does your stewardship program monitor compliance with one or more of the specific interventions in place?		
**ANTIBIOTIC USE AND OUTCOME MEASURES**		
Does your facility track rates of *C. difficile* infection?		
Does your facility produce an antibiogram (cumulative antibiotic susceptibility report?)		
Does your facility monitor antibiotic use (consumption) at the unit and/or facility wide level by one of the following metrics?		
By counts of antibiotic(s) administered to patients per day (Days of Therapy; Directly Observed Therapy)?		
By number of grams of antibiotics used (Defined Daily Dose (DDD), Anatomical Therapeutic Classification)?		
By direct expenditure for antibiotics (purchasing costs)?		
Other, Specify:		

5. REPORTING INFORMATION TO STAFF ON IMPROVING ANTIBIOTIC USE	Yes	No
Does your stewardship program share facility-specific reports on antibiotic use with prescribers?		
Has a current antibiogram been distributed to prescribers at your facility?		
Do prescribers ever receive direct, personalized communication about how they can improve their antibiotic prescribing?		
Other, Specify:		

6. EDUCATION	Yes	No
Does your stewardship program provide education to clinicians and other relevant staff on improving antibiotic prescribing?		
Have any of the members of the AMS team attended AMS training?		
Other, Specify:		

CHALLENGES EXPERIENCED IN THE IMPLEMENTATION OF AMS ACTIVITIES		Tick All that Apply
Lack of management support		
Lack of human resources:	Clinicians	
	Pharmacists	
	Nurses	
No access to a microbiologist		
Lack of time		
Lack of training		
Lack of printed antibiotic guidelines		
Financial restrictions regarding microbiological investigations		
Lack of multidisciplinary approach		
Lack of clinician buy in		
Other, Specify:		

SECTION D

If No functional Antimicrobial Stewardship committee exists at your hospital, what are the barriers to the establishment of an active AMS committee?

**Activities**		**Tick All that Apply**
Lack of management support		
Lack of expertise e.g. no microbiologist		
Lack of time		
Lack of training		
Lack of printed antibiotic guidelines		
Financial restrictions regarding microbiological investigations		
Lack of multidisciplinary approach		
Lack of clinician buy in		
Lack of human resources:	Clinicians	
	Pharmacists	
	Nurses	
Other challenges, Specify:		

Pharmaceutical Services appreciates your input. The information will be used to strengthen AMS activities in our province.

Acknowledgements

This survey has been prepared by combining an adapted Center for Disease Control (CDC)[1] and Department of Health (DOH) survey tools. Permission was obtained from the CDC and DOH for use of these tools.

S 3

Feedback on Antimicrobial Stewardship (AMS) Survey

Thank you for assisting the KwaZulu-Natal (KZN) Antimicrobial Stewardship (AMS) Committee (KZN-AMSC) to pilot an AMS situational analysis survey developed for use in KZN. The feedback on your experience completing the survey is valued as it will help the KZN-AMSC improve the survey tool before dissemination to the rest of the province.

Please provide feedback on the AMS survey by indicating your agreement with the statements in the following table, by placing a tick in the appropriate cell.

	**Strongly Agree**	**Agree**	**Neutral**	**Disagree**	**Strongly Disagree**
**The survey covered the main aspects of Antimicrobial Stewardship**					
**The survey was easy to understand**					
**The survey was easy to complete**					
**The instructions accompanying the survey were clear and understandable**					
**The length of time it took me to complete the survey was appropriate**					

	Yes	No
1. Would the facility have preferred an internet-based tool (e.g. Survey Monkey)		

2. How long did it take you to complete the survey?

……

3. List questions that were of concern/too long, short/other. Please provide details and reasons for your observations:
………
………
………
………
………

4. Please provide any additional comments or suggestions to improve the survey:

………
………
………
………
………
………
………
……

Thank You

References

1. CDC. Core Elements of Hospital Antibiotic Stewardship Programs. Atlanta, GA: US Department of Health and Human Services, CDC; . *Available at http://wwwcdcgov/getsmart/healthcare/implementation/core-elementshtml* 2014.

Table S1 Bivariate Chi-squared test of association and Multivariable logistic regression: Associations between Drug and Antimicrobial Expertise (clinical microbiologist)/ Drug and Antimicrobial Expertise (Infectious disease physician)/ Composition of the Antimicrobial Stewardship (clinical microbiologist) and different AMS Interventions

Interventions	Drug & Antimicrobial Expertise, n (%)			aOR(95%CI)	aOR p-value
	Is there a clinical microbiologist on site or is there off-site support from a clinical microbiologist		Chi-square p-value		
	Yes	No			
**Microbiologist input on pathogen surveillance data**					
Yes	11(84.6)	2(15.4)	**0.000****	**5.12(4.08-22.02)**	**0.001****
No	5(16.7)	25(83.3)		1	
**Microbiological investigations prior to**					
Yes	10(62.5)	6(37.5)	**0.011***	**6.73(1.08-42.01)**	**0.041***
No	6(23.1)	20(76.9)			
	**Composition of the Antimicrobial Stewardship Committee, n (%)**			**aOR(95%CI)**	**aOR p-value**
	**Is there a representative from microbiology**		**Chi-square p-value**		
	**Yes**	**No**			
**Availability of AMR**					
Yes	12(54.5%)	10(45.5%)	0.051*	3.21(0.22-46.52)	0.389
No	5(25%)	15(75%)		1	--
**Interrogation of AMR**					
Yes	9(60%)	6(40%)	0.036*	0.51(0.03-9.18)	0.647
No	7(26.9%)	19 (73.1%)		1	
**Microbiologist input on pathogen surveillance data**					
Yes	12(92.3)	1(7.7%)	**0.000****	**43.54(4.03-147.65)**	**0.002**
No	5 (17.2%)	24 (82.8%)		1	
**Microbiological investigations prior to**					
Yes	8(50%)	8(50%)	0.375	---	---
No	9(36.0%)	16(64%)		---	---
**Review of antibiotic with culture results**					
Yes	14(45.2%)	17 (54.8%)	0.397	---	---
No	3 (30%)	7(70%)		---	---
	**Drug and Antimicrobial Expertise, n (%)**			**aOR(95%CI)**	**aOR p-value**
	**Is there an infectious disease physician on site**		**Chi-square p-value**		
	**Yes**	**No**			
**Microbiological investigations prior to**					
Yes	2(12.5%)	14(87.5%)	0.065	---	---
No	0(0%)	26(100%)		---	---
**Empiric treatment in line with Standard Treatment**					

Yes	2(5%)	38(95%)	0.692	---	---
No	0(0%)	3(100%)		---	---
**Documented Indication for antibiotics**					
Yes	2(5.4%)	35(94.6%)	0.594	---	---
No	0(0%)	5(100%)		---	---
**Review of antibiotic with culture results**					
Yes	2(6.5%)	29(93.5%)	0.410	---	---
No	0(0%)	10(100%)		---	---
**Change in antibiotic prescribing – cease therapy / de-escalation / substitution /**					
Yes	1(3%)	32(97%)	0.313	---	---
No	1(11.1%)	8(88.9%)		---	---

Key: aOR: adjusted odds ratio; Statistical significance: (*)P <0.05 and (**)P <0.01

Table S2Bivariate Chi-squared test of association and Univariate logistic regression: Associations between Drug and Antimicrobial Expertise (clinical microbiologist)/ Composition of the Antimicrobial Stewardship (clinical microbiologist) and Tracking: monitoring antibiotic, prescribing, use, resistance

Bivariate Chi-squared test of association and Multivariable logistic regression: Associations between Drug and Antimicrobial Expertise (Infectious disease physician and Tracking: monitoring antibiotic, prescribing, use, resistance

**Tracking: monitoring antibiotic , prescribing, use, resistance**	**Drug & Antimicrobial Expertise, n (%)**			**aOR(95%CI)**	**aOR p-value**
	**Is there a clinical microbiologist on site or is there off-site support from a clinical microbiologist**		**Chi-square p-value**		
	**Yes**	**No**			
**Does your facility track rates of *C. difficile***					
Yes	7(50%)	7(50%)	0.231	2.25(0.59-8.58)	0.235
No	8(30.8%)	18(69.2%)		1	
	**Composition of the Antimicrobial Stewardship Committee, n (%)**			**aOR(95%CI)**	**aOR p-value**
	**Is there a representative from microbiology**		**Chi-square p-value**		
	**Yes**	**No**			
**Does your facility produce an antibiogram**					
Yes	12(60%)	8(40%)	0.019**	**4.80(1.25-18.42)**	**0.022***
No	5(23.8%)	16(76.2%)		1	
	**Drug and Antimicrobial Expertise, n (%)**			**aOR(95%CI)**	**aOR p-value**
	**Is there an infectious disease physician on site**		**Chi-square p-value**		
	**Yes**	**No**			

Does your stewardship program monitor adherence to facility specific treatment guidelines					
Yes	2(10%)	18(90%)	0.129	---	---
No	0(0%)	22(100%)		---	---
**Does your stewardship monitor compliance with one or more of the specific interventions in place**					
Yes	2(8.3%)	22(91.7%)	0.209	---	---
No	0(0%)	18(100%)		---	---

Key: aOR: adjusted odds ratio; Statistical significance: (*)P <0.05 and (**)P <0.01

Table S3 Bivariate Chi-squared test of association and Univariate logistic regression: Association between composition of the Antimicrobial Stewardship (clinical microbiologist) and distribution of current antibiogram.

Bivariate Chi-squared test of association and Multivariable logistic regression: Associations between Drug and Antimicrobial Expertise (Infectious disease physician)

Reporting information to staff on improving antibiotic use	Composition of the Antimicrobial Stewardship Committee, n (%)			aOR(95%CI)	aOR p-value
	Is there a representative from microbiology		Chi-square p-value		
	Yes	No			
**Has a current antibiogram been distributed to**					
Yes	5(50%)	5(50%)	0.529	1.58(0.38-6.45	0.530
No	12(38.7%)	19(61.3%)		1	--
	**Drug and Antimicrobial Expertise, n (%)**			**aOR(95%CI)**	**aOR p-value**
	**Is there an infectious disease physician on site**		**Chi-square p-value**		
	**Yes**	**No**			
**Does your stewardship share facility-specific reports on antibiotic use**					
Yes	2(6.5%)	29(93.5%)	0.388	---	---
No	0(0%)	11(100%)		---	---
**Has a current antibiogram been distributed to**					
Yes	2(20%)	8(80%)	0.010**	---	---
No	0(0%)	32(100%)		---	---

**Do prescribers ever receive direct, personalized communication about how they can improve their**					
Yes	0 (0%)	27(100%)	0.044*	---	---
No	2(14.3%)	12(85.7%)		---	---

Key: aOR: adjusted odds ratio; Statistical significance: (*) P <0.05 and (**)P <0.01

Table S4 Bivariate Chi-squared test of association and Multivariable logistic regression: Associations between Drug and Antimicrobial Expertise (Pharmacist Leader) and Interventions, Tracking (Monitoring antibiotic prescribing, use and resistance) and reporting information to staff on improving antibiotic use.

Interventions	Drug & Antimicrobial Expertise, n (%)			aOR(95%CI)	aOR p-value
	Is there a pharmacist leader		Chi-square p-value		
	Yes	No			
**Documented indication for antibiotics**					
Yes	36(97.3%)	1(27%)	**0.710**	**---**	**---**
No	5(100%)	0(0%)		---	---
**Review of antibiotic with culture results**					
Yes	31 (100%0	0(0%)	**0.075**	**----**	**---**
No	9(90%)	1(10%)		---	---
**Change in antibiotic prescribing – cease therapy / de-escalation / substitution / addition of agents**					
Yes	32(97%)	1(3%)	**0.597**	---	---
No	9(100%)	0(0%)		---	---
**Is there a formal procedure for all clinicians to review the appropriateness of all antibiotics 48 hours after the initial prescription orders (e.g. antibiotic time out)**					
Yes	14(100%)	0(0%)	**0.314**	---	---
No	27(93.1%)	2(6.9%)		---	---
**Intravenous (IV) to oral**					
Yes	30(100%)	0(0%)	**0.079**	---	---
No	9(90%)	1(10%)		---	---
**Hang time**					
Yes	21(100%0	0(0%)	**0.274**	---	---
No	17(94.4%)	1(5.6%)		---	---
**Are there dedicated pharmacy ward rounds?**					

Yes	24(100%)	0(0%)	**0.094**	---	---
No	16(88.9%)	2(11.1%)		---	---
**Dose adjustments in cases of organ dysfunction**					
Yes	34(97.1%)	1(2.9%)	**0.195**	---	---
No	6(85.7%)	1(14.3%)		---	---
**Drug interactions**					
Yes	37(97.4%)	1(2.6%)	**0.776**	---	---
No	3(100%)	0(0%)		---	---
**Dose optimization**					
Yes	30(100%)	0(0%)	**0.028**	---	---
No	11(84.6%)	2(15.4%)		---	---
**Automatic alerts in situations where therapy might be unnecessarily duplicative**					
Yes	18(100%)	0(0%)	**0.200**	---	---
No	21(91.3%)	2(8.7%)		---	---
**Time-sensitive automatic stop orders for specified antibiotic prescriptions**					
Yes	12(100%)	0(0%)	**0.351**	---	---
No	27(93.1%)	2(6.9%)		---	---

**Tracking: monitoring antibiotic , prescribing, use, resistance**	**Drug & Antimicrobial Expertise, n (%)**			**aOR(95%CI)**	**aOR p-value**
	**Is there a pharmacist leader**		**Chi-square p-value**		
	**Yes**	**No**			
**Does your stewardship program monitor adherence to a documentation policy (dose, duration, and indication)**					
Yes	26(100%)	0(0%)	**0.073**	---	---
No	15(88.2%)	2(11.8%)		---	--
**Does your stewardship program monitor adherence to facility-specific treatment recommendations**					
Yes	20(100%)	0(0%0	**0.167**	---	---
No	20(90.9%)	2(9.1%)		---	---
**Does your stewardship program monitor compliance with one or more of the specific interventions in place?**					
Yes	24(100%)	0(0%)	**0.094**	---	---

No	16(88.9%)	2(11.1%)		---	---
**By counts of antibiotic(s) administered to patients per day (Days of Therapy; Directly Observed Therapy)**					
Yes	14(100%)	0(0%)	**0.314**	---	---
No	27(93.1%)	2(6.9%)		---	---
**By number of grams of antibiotics used (Defined Daily Dose (DDD)**					
Yes	10(100%)	0(0%)	**0.425**	---	---
No	31(93.9%)	2(6.1%)		---	---
**By direct expenditure for antibiotics (purchasing costs)**					
Yes	26(100%)	0(0%)	0.073	---	---
No	15(88.2%)	2(11.8%)		---	---

**Reporting information to staff on improving antibiotic use**	**Drug and Antimicrobial Expertise, n (%)**			**aOR(95%CI)**	**aOR p-value**
	**Is there a pharmacist leader**		**Chi-square p-value**		
	**Yes**	**No**			
**Does your stewardship program share facility-specific reports on antibiotic use with prescribers?**					
Yes	30(96.8%)	1(3.2%)	0.547	---	---
No	11(100%)	0(0%)		---	---
**Do prescribers ever receive direct, personalized communication about how they can improve their antibiotic prescribing**					
Yes	27(100%)	27(100%)	No statistics are computed because Is there a pharmacist leader responsible for improving antibiotic use at the facility is a constant	---	---
No	14(100%)	14(100%)		---	---

Key: aOR: adjusted odds ratio; Statistical significance: (*)P <0.05 and (**)P <0.01
